# Clinical Management of Endometriosis in Menopause: A Narrative Review

**DOI:** 10.3390/medicina60081341

**Published:** 2024-08-18

**Authors:** Dhruva Dave, Heidi E. Page, Aakriti R. Carrubba

**Affiliations:** 1 Gujarat Medical Education and Research Society (GMERS), Medical College and Hospital, Vadodara 390021, India; 2 Department of Medical and Surgical Gynecology, Mayo Clinic, Jacksonville, FL 32224, USA

**Keywords:** endometriosis, menopause, endometriosis-associated ovarian cancer, de novo endometriosis, hormone replacement therapy

## Abstract

Endometriosis, an inflammatory disease primarily affecting the pelvis and peritoneum, manifests with pelvic pain, dysmenorrhea, dyschezia, dyspareunia, and infertility. Despite its ubiquity, the management of endometriosis is challenging due to its heterogeneous presentation, limitations in diagnostic methods, variable therapeutic responses, and personal and socio-cultural impact on quality of life. This review attempts to consolidate the current literature on endometriosis occurring during and beyond menopause, and to present details regarding management strategies that take into account individual outcomes and goals when managing this condition. The topics included in this review are the clinical features and differential diagnosis of pelvic pain in postmenopausal patients, imaging considerations, serum and laboratory biomarkers, indications for surgery, the principles of hormone replacement therapy, the de novo development of endometriosis after menopause, and malignant transformation. Each topic includes a summary of the current literature, utilizing clinical research, case reports, and expert opinion. Despite a better understanding of the impact of endometriosis beyond menopause, there are many limitations to this condition, specifically with regard to cancer risk and indications for surgery. The existing evidence supports the use of shared decision making and the incorporation of patient preferences in guiding clinical management. Future research endeavors must shed light on the natural history of postmenopausal endometriosis through longitudinal studies in order to foster a deeper understanding of its complicated disease course across women’s lifespans.

## 1. Introduction

Endometriosis classically affects reproductive-aged women, and is associated with pelvic pain, dysmenorrhea, dyschezia, dyspareunia, and infertility. This inflammatory disease is marked by ectopic endometrial glands and stromal tissue scattered in the pelvis and governed by the presence of estrogen. Endometriosis is associated with pelvic pain, dysmenorrhea, infertility, dyspareunia, impact on sexual relationships, lost wages, work absenteeism, and disability [1,2,3]. Despite its prevalence, there are many barriers to the delivery of endometriosis care due to variable disease presentation, the poor sensitivity of non-invasive diagnostic testing, variable surgeon experience and skill, chronic overlapping pain conditions, variable response to hormone therapy, the invalidation of the patient’s experience, poor health literacy, and social media emphasis on radical surgery [4,5,6,7]. Assessing the impact of symptoms on various aspects of daily life, including physical functioning, emotional well-being, and social interactions, provides a holistic understanding of symptom severity [7].

The response to previous medical and surgical treatments for endometriosis should be carefully evaluated for the specific patient to guide the subsequent management decisions owing to unique genetic makeup and environmental factors. Medical therapies such as nonsteroidal anti-inflammatory drugs (NSAIDs), hormonal contraceptives, gonadotropin-releasing hormone (GnRH) agonists, and progestins may have been used to manage symptoms and control disease progression. Surgical interventions, including the laparoscopic excision of endometriotic lesions, may have been performed to alleviate symptoms and improve fertility outcomes [8].

A systematic review recently demonstrated the importance of hormonal suppression following surgical excision to reduce the likelihood of recurrent lesions and pain [9]. When the uterus remains in place, the placement of a levornorgestrel intrauterine device (LNG IUD) can be beneficial. A Cochrane review including three randomized controlled trials demonstrated a statistically significant reduction in painful menses in LNG IUD users compared with expectant management, as well as lower pain scores compared to GnRH users [10]. Other studies have shown no significant improvement in dysmenorrhea, quality of life, and overall patient satisfaction due to the heterogeneous nature of the published studies on endometriosis and overall low-quality evidence [11]. The placement of LNG IUD should be individualized and offered as a trial for patients who are interested in this therapy, with an understanding that the effects are variable. Some patients do not respond to typical hormone treatments, and it is postulated that up to one-third of women may exhibit progesterone resistance within endometriotic lesions. This may be related to the interactions of the progesterone receptor (PR) A and PR-B isoforms, which are functionally different and have downstream physiologic effects to activate and inhibit the effect of progesterone on peripheral tissue [12]. A systematic review on this topic found that deep endometriosis lesions tend to be more resistant to progestins which may be related to oxidative stress and the predominance of PR-A, the less active isoform [13]. With this in mind, it is still important to offer and discuss hormone suppression following surgery, but to consider alternative options such as GnRH agonists or selective progesterone receptor modulators in cases where progesterone resistance is suspected.

Menopause is defined as the permanent cessation of ovarian function and the end of a woman’s reproductive potential, and with it comes a drastic reduction in systemic estrogen levels [14]. For this reason, many clinicians counsel patients that endometriosis-related pain and symptoms improve following menopause. Furthermore, treatments that induce a hypoestrogenic state such as gonadotropin-releasing hormone (GnRH) agonists and antagonists have been a mainstay of the medical management of endometriosis, and may provide pain relief in a subset of patients [15,16,17]. A Cochrane review from 2022 including 72 articles found a slight decrease in overall pain in patients using GnRH analogs compared to placebo or oral or injectable progestogens, but the quality of evidence was low due to the heterogeneity of the studies [16]. 

Endometriosis may continue to be a concern in postmenopausal patients. The prevalence of endometriosis in menopause is estimated to be 2–4% based on the literature [8,18,19]. It can be associated with persistent pain, and the alteration of anatomy in cases of deep infiltrating disease can impact choices for hormone replacement therapy, and rarely can undergo malignant transformation. It is also important to note that the onset of menopause may be earlier in women with endometriosis. This was found in a population-based cohort study including over 100,000 premenopausal women from 1989 to 2015, of which 3921 had surgically confirmed endometriosis. Laparoscopically confirmed endometriosis was associated with a 50% greater risk for early natural menopause (defined as 12 consecutive months of amenorrhea prior to the age of 45). There are different theories as to why this occurs, but it may be related to diminished ovarian reserve from the presence of endometriomas, oxidative stress, and inflammatory mediators within the peritoneal fluid [20].

The objective of this review article is to perform and present a literature search of the diagnostic considerations, management, and malignant potential of postmenopausal endometriosis in order to outline management strategies for clinician support.

## 2. Diagnosis

### 2.1. Evaluation of Postmenopausal Pelvic Pain

One of the hallmark symptoms of endometriosis is pelvic pain. However, it is important to keep a broad differential diagnosis as there are many causes of chronic pelvic pain. Postmenopausal patients may have pain related to the genitourinary syndrome of menopause, pelvic floor tension myalgia, pudendal neuralgia, pelvic congestion syndrome, vulvodynia, endometriosis, and adnexal masses, among others (summarized in Table 1) [21]. A detailed history including pain characteristics, the time of onset, relieving and exacerbating factors, and associated symptoms is essential. This should be followed by abdominal and pelvic examination to evaluate the external genitalia, vestibule, assess pelvic floor musculature, ischial spines, and the evaluation of the vaginal vault [22,23]. Pelvic exam findings that may raise suspicion for endometriosis-related pain include the nodularity and tenderness of the uterosacral ligaments, a fixed or deviated cervix, and/or painful adnexal masses.

Post-menopausal patients with a known history of endometriosis may have had surgeries in the past, including hysterectomy or bilateral salpingo-oophorectomy (BSO). Pain along the vaginal cuff can be caused by adhesion formation, alterations in the innervation of the vagina, or the formation of a neuroma [24,25]. Additionally, studies have shown that vaginal hysterectomy is associated with significantly shorter vaginal length, and dyspareunia can result from a reduction in canal length [26]. The careful assessment of the cuff utilizing a Q-tip can identify trigger points and tenderness.

Ovarian remnant syndrome is another condition that may occur in this population. It is defined by the presence of residual ovarian tissue in patients who have undergone prior unilateral or bilateral oophorectomy [27]. Ovarian remnant tissue can produce estradiol, and clinical suspicion can be raised in premenopausal patients who fail to experience classic vasomotor symptoms after BSO. Definitive diagnosis occurs at the time of surgical exploration with histological confirmation, and treatment consists of the excision of all remaining ovarian tissue [28].

### 2.2. Imaging of Endometriosis

Imaging for endometriosis is challenging and requires a high index of suspicion. Ultrasound is often the first-line modality to evaluate pelvic anatomy. In premenopausal women with the classic symptoms of dysmenorrhea and cyclical pain, findings of an adnexal mass with homogenous echotexture or abnormal sliding sign can reliably indicate deep endometriosis of the ovaries and posterior compartment [29,30]. In a postmenopausal patient without prior comparison imaging, adnexal masses may be more suspicious for malignancy and less likely for the clinician to suspect an endometrioma.

Pelvic magnetic resonance imaging (MRI), particularly if an endometriosis protocol is used (vaginal gel, octreotide for halting bowel peristalsis, and rectal enemas), may be more sensitive for the evaluation of deep infiltrating endometriosis. The typical features of ovarian endometriomas include solitary or multiple masses with homogenous hyperintense signal on T1-weighted images and variability of density with a thick, hypointense fibrous capsule on T2-weighted images [31]. These findings help to reliably differentiate an endometrioma from other types of adnexal masses. The MRI features of deep infiltrating disease include T2 hyperintense foci, solid nodules, plaque-like thickening, or a stellate or spiculated lesion involving the soft tissue [31]. Additionally, the evaluation of the soft tissue of the pelvis can evaluate for lesions involving the bladder and rectum, and identify concerns regarding the obliteration of the posterior cul-de-sac.

**Figure 1:**
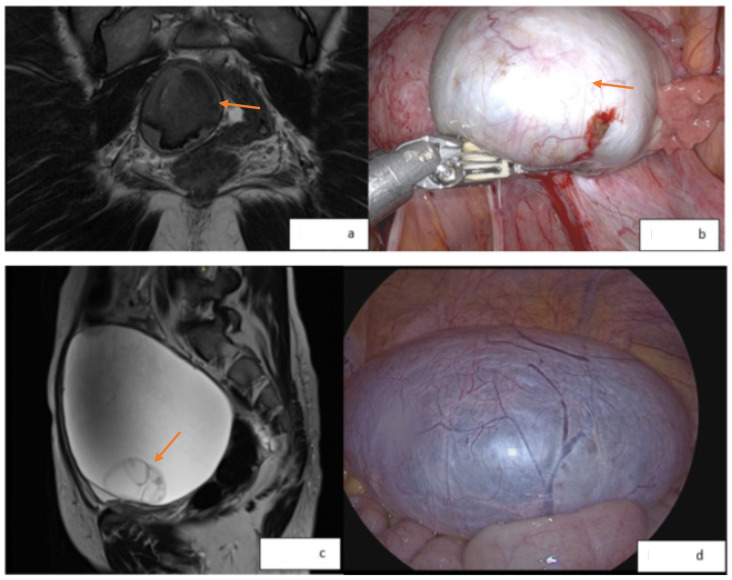
(**a**): O-RADS 2 lesion; 4.7 cm right ovarian endometrioma (orange arrow) with no enhancement, nodularity, or suspicious features. (**b**): Laparoscopic image (orange arrow indicating lesion); pathology consistent with endometriosis. (**c**): O-RADS 3 lesion; increasing size and complexity of a right adnexal mass measuring 13.5 cm (orange arrow indicating the areas of septations). (**d**): Laparoscopic image; pathology consistent with the grade 1 endometrioid adenocarcinoma of the ovary.

There is also an additional ability to evaluate for atypical features within an endometrioma. The MRI features of endometriosis-associated ovarian cancer (EAOC) include intermediate T2 signal (particularly the loss of T2 shading due to the dilution of hemosiderin within an endometrioma by increased fluid produced by a tumor), solid enhancing mural nodules within an otherwise cystic lesion, irregular and nodular septations, and restricted diffusion on diffusion-weighted imaging (DWI) [31,32]. It is important to remember that these findings are non-specific and may not always be associated with malignancy [33]. Examples of MR images and associated laparoscopic findings from normal and malignant endometriomas are shown in Figure 1.

There are multiple scoring tools which use clinical and imaging criteria to determine the risk of malignancy within adnexal masses. Some examples include the gynecologic imaging reporting and data system (GI-RADS), the international ovarian tumor analysis (IOTA) simple rules, and a newer tool called the ovarian–adnexal reporting and data system (O-RADS) classification system [7]. This system was developed by the American College of Radiology (ACR) via a committee comprising experts with the objective of assigning a probability of malignancy based on the ultrasound or MRI features of an adnexal lesion; it has similar inter-reviewer agreement with GI-RADS and IOTA simple rules, but has a higher sensitivity for malignancy [34,35]. A variety of features, including patient age, lesion size, and characteristics of the mass are used in an algorithm to provide an O-RADS score with the associated cancer risk (Table 2). A systematic review by Rizzo et al. demonstrated a 92% sensitivity and 91% specificity of the MRI O-RADS score with final pathology, with a 60% malignancy rate in O-RADS 4 and 96% malignancy rate in O-RADS 5 lesions [36]. Another systematic review showed that the pooled sensitivity and specificity of O-RADS ultrasound-based system were 95% (95% CI, 91–97%) and 82% (95% CI, 76–87%), and of O-RADS MRI-based system were 95% (95% CI, 92–97%) and 90% (95% CI, 84–94%) [37]. These imaging modalities and scoring systems are key for counseling and surgical decision making, including appropriate referrals to gynecologic oncology based on clinical suspicion.

### 2.3. Laborotory Assessment: Biomarkers

Endometriosis is diagnosed with histologic assessment, and often requires laparoscopic surgery for confirmation. There are many patients with classic symptoms of endometriosis who want to avoid surgery, and there has been much interest in the role of biomarkers and laboratory assessment to assist with diagnosis.

Tumor markers, such as Cancer Antigen 125 (Ca-125), are important tools in both the diagnosis and management of endometriosis among postmenopausal women. Their utility becomes particularly pronounced in identifying advanced stage III or IV endometriosis [40]. Normal values of Ca-125 are different in premenopausal women (<200 units/mL) and postmenopausal women (<35 units/mL). This lab test is widely recognized as a hallmark marker for ovarian cancer, but can also be elevated in various benign gynecologic disorders, including endometriosis [41]. In the postmenopausal population, the assessment of Ca-125 with complementary markers like Human Epididymis protein 4 (HE4) and Carcinoembryonic Antigen (CEA) enhances both the sensitivity and specificity of tumor marker accuracy in the diagnostic process. This not only aids in pinpointing endometriosis but also serves to differentiate it from potentially malignant conditions [42]. HE4 is a whey acid protein that is expressed in the female reproductive tract and is over-expressed in patients with serous and endometrioid epithelial ovarian cancers, and is not expressed in normal ovarian tissue [43]. Moreover, the utilization of a comprehensive panel of serum markers, including Ca-125 and interleukin 6 and 8, has yielded promising outcomes in the diagnosis of mild to moderate endometriosis [43]. It is important to note that Ca-125 assessment should be interpreted with caution. A Cochrane review from 2016 found varying sensitivities and specificities for different cut-off values of Ca-125 for endometriosis, and concluded that there was insufficient evidence for any blood test to replace laparoscopy in the formal diagnosis of endometriosis due to lack of accuracy [44].

Recently, new and promising diagnostic methods for the detection of endometriosis have emerged. One example includes salivary microRNA, which are single-stranded, highly conserved, non-coding RNAs that regulate gene degradation and translation when they bind. The ENDO-miRNA study included 200 saliva samples obtained from women with chronic pelvic pain suggestive of endometriosis, and found that 76.5% of the patients with a diagnostic miRNA signature were actually diagnosed with endometriosis [45]. Additional multi-center trials are being performed to validate the results of the ENDO-miRNA study to further develop the signature using next-generation sequencing and artificial intelligence, and have shown a sensitivity of 96.2% and specificity of 95.1% [46].

## 3. Indications for Surgery

Surgical intervention for postmenopausal women with endometriosis spans a wide range of indications, primarily the anatomical concerns like ureteral or bowel obstruction due to endometriosis lesions; however, surgical management may also be necessary for symptomatic relief or managing complications arising from pelvic adhesions, endometriomas, or deep infiltrating endometriosis [47]. Furthermore, the presence of pelvic pain resistant to medical therapy or the development of endometriosis-associated complications such as ovarian torsion or rupture further poses the need for surgical intervention [47]. Additionally, refractory urinary symptoms such as hematuria, recurrent urinary tract infections, or hydronephrosis secondary to ureteral involvement by endometriosis may require surgical exploration to alleviate obstruction and prevent further complications [48]. The identification of suspicious adnexal masses, as described in the section on imaging above, may also prompt surgical exploration to rule out malignancy, especially relevant in postmenopausal women.

Surgery plays a crucial role in managing postmenopausal endometriosis by offering symptom relief and the enhancement of overall quality of life through the normalization of anatomy. Studies have shown that surgery can effectively address chronic pelvic pain and dyspareunia in a large majority of patients [49]. If uterine disorders are present, such as postmenopausal bleeding, fibroids, or adenomyosis, a hysterectomy can be performed concurrently. The standard of care for surgical intervention in endometriosis is minimally invasive laparoscopy, given the improved post-operative and intra-operative outcomes, but laparotomy may be indicated in the cases of malignant transformation to ovarian cancer (discussed below). These considerations explain the importance of personalized care and a customized approach in the management of postmenopausal women with endometriosis. However, not all patients require surgical intervention, especially if they are asymptomatic; do not have worrisome features on imaging; or are not appropriate surgical candidates due to medical comorbidities. Surgery is associated with risks, such as pain, bleeding, infection, damage to surrounding structures, anesthetic-related complications, and prolonged recovery. The decision for surgical intervention should be made to align with the patient’s goals and include a careful consideration of risks and benefits. If expectant management is selected, there are no published guidelines for screening and follow-up. In our practice, we recommend annual gynecologic examinations and the consideration of imaging based on clinical changes.

## 4. Hormone Replacement Therapy

Hormone Replacement Therapy (HRT) is the cornerstone in the management of systemic symptoms of menopause, including mood fluctuations, sleep disruptions, cognitive challenges, and vasomotor symptoms (VMSs) like hot flashes, while preventing osteoporosis-related fractures and optimizing cardiovascular health. This can be administered via estrogen alone or combined estrogen and progestin formulations, and the addition of progestin is typically indicated in women with an intact uterus to prevent endometrial hyperplasia. However, the use of HRT in postmenopausal women with endometriosis is challenging and controversial.

The hypothalamic thermoregulatory neutral zone regulating the basal body temperature is physiologically inhibited by estrogen. During the estrogen withdrawal in the menopausal period, these neurons become overstimulated for the co-expression of kisspeptin, neurokinin B, and dynorphin. This eventually leads to dysregulated body temperature manifested as bothersome vasomotor symptoms like hot flashes, skin reddening, warmth, and perspiration [50]. Considering the high prevalence and significant impact on quality of life, HRT remains the mainstay of treatment strategies for VMS in postmenopausal women. The benefits of HRT outweigh the risks for healthy patients under age 60 within 10 years of their first menstrual period [51]. The risk of venous thromboembolism (VTE) is highest immediately after starting HRT and drastically reduces to the baseline level of risk after discontinuation. Furthermore, the risk of VTE may vary based on the type of estrogen; a population-based case–control study found a statistically significant increased risk of VTE with conjugated equine estrogen compared with estradiol therapy alone [52]. Additionally, there is bias and fear of the risk of breast cancer associated with HRT use. The Women’s Health Initiative Estrogen Plus Progestin Trial in 2004 randomized 16,608 postmenopausal women with a uterus to a combination of daily oral conjugated equine estrogen 0.625 mg/d and medroxyprogesterone acetate 2.5 mg/d or placebo and was abruptly stopped due to the increased incidence of breast cancer in the treatment population [53]. However, estrogen-only formulations like estradiol 17β have shown significant breast cancer mortality reduction in postmenopausal women. The National Finnish Comparative Study observational trial demonstrated that breast cancer mortality reduction in women using isomolecular estradiol was much more compared with a control group with no HRT [54]. This trend was also replicated when equine estrogen alone was administered to the postmenopausal versus the placebo group [55].

One key concern is the effect of unopposed estrogen, which may exacerbate endometriosis-associated symptoms due to estrogen’s role in promoting the growth and proliferation of endometrial tissue. A study by Guo et al. highlighted that unopposed estrogen therapy increased the risk of endometriosis recurrence in postmenopausal women [14]. The addition of progesterone has been proposed to mitigate this risk by counteracting the proliferative effects of estrogen on endometrial tissue. In a randomized controlled trial, Vercellini et al. demonstrated that combined estrogen–progesterone therapy reduced pelvic pain and lesion size in postmenopausal women with endometriosis compared to estrogen-alone therapy [56]. However, the reactivation of endometriotic lesions with HRT remains a subject of debate, with some studies suggesting an increased risk of symptom recurrence with hormone therapy, while others report no significant impact [57,58]. Furthermore, the association between HRT and endometriosis-related pain in postmenopausal women warrants careful consideration. While some studies suggest a potential exacerbation of pain symptoms with HRT, others indicate no significant increase in pain severity [59,60].

The reactivation of endometriosis implants in postmenopausal women indicates the persistent influence of estrogen on endometrial tissue. The occurrence of de novo endometriosis highlights the multifactorial nature of the disease involving both hormonal and inflammatory pathways. In both these cases, a combined hormone replacement therapy approach incorporating both estrogen and progesterone offers a promising strategy for managing endometriosis in this population to essentially ameliorate their quality of life. Overall, the decision to initiate HRT in postmenopausal women with endometriosis necessitates a personalized approach, considering the following factors and a balance between symptom relief and potential disease exacerbation.

## 5. De Novo Endometriosis Development

The drastic estrogen depletion during menopause supports the belief that de novo endometriosis in menopause is a rare possibility. However, this belief was questioned by the groundbreaking case of endometriosis reported by Edgar Haydon in 1942 in a postmenopausal patient [18,61]. Since that time, there have been numerous reports of endometriosis in postmenopausal women. Some studies report an association of estrogen-alone hormone replacement therapy as the primary risk factor for endometriosis development [62]. However, there have been other reports of endometriosis arising in the absence of identifiable risk factors, including patients not using exogenous hormones.

Table 3 shows some examples of the published cases of primary endometriosis occurring in postmenopausal patients, which were obtained by searching the terms “postmenopausal” and “de novo endometriosis” in the Medline database. While these reports are not all-encompassing, we have included this table to depict the presentation and management of this disease process. It is worth mentioning that endometriosis may have been present prior to menopause in these cases, and without baseline imaging or prior surgical assessment of the pelvis, it is impossible to know whether these cases truly occurred de novo or were indolent and asymptomatic.

## 6. Malignant Transformative Potential

Endometriosis is thought to be a premenopausal benign condition, but there is a potential for transformation to associated gynecologic cancers. Specifically, endometriosis is associated with ovarian endometrioid carcinoma and epithelial ovarian cancers such as clear cell, low-grade serous, and mixed cell, which are termed endometriosis-associated ovarian cancer (EAOC). Although the pathophysiology of endometriosis evolution is controversial, one proposed mechanism is via the regurgitation of tubal and uterine epithelium through the fallopian tubes to the pelvic cavity [77]. This would then increase the risk of neoplastic transformations if uterine cell migrations and implant to the ovary [78]. Another theory purports that cancer develops due to the hyperplasia of endometrial glands with cytological atypia or the presence of atypical hobnail cells within ovarian endometriosis, especially since atypical endometriosis can be found in up to 80% of the cases of EAOC [79]. Criteria defining malignant transformation of endometriosis include the location of endometriosis close to the tumor, malignant foci arising within endometrioid lesions, and findings of a transitional area showing the progression from benign to malignant disease [80]. The overall risk of EAOC in patients with known endometriosis is 2–3% [47,79]. Despite this low incidence, it is imperative to educate patients about potential risk factors to continue surveillance measures, including pelvic exams, tumor maker evaluations, and imaging. 

Endometriosis and EAOCs share similar risk factors including the early onset of menarche, nulliparity, and estrogen dependencies. Endometrial cancer and endometriosis share clinicopathological characteristics as they both cause inflammation, invasion, and resistance to apoptosis as well as the stimulation of angiogenesis [81]. Genetic alterations have been linked to ovarian malignancy, specifically PTEN gene missense mutation/deletions and ARID1A mutations [78]. The ARID1A-encoded protein and BAF250a expression genes are most associated with clear cell and endometrioid carcinomas as well as benign endometriosis. This is thought to be caused by DNA reconstruction or “damage” causing the breakdown of interactions and activation pathways for lesion formations. The ARID1A gene has been found in 41–57% of clear cell carcinomas and 30–48% of endometrioid cancers [82]. Additional mutations exist in the CTNNB1, PIK3CA, KRAS, TP53, and SOX8 genes in endometrioid ovarian cancers, which may have impacts on clinical outcomes [83].

A systematic review of 75 studies including 90 unique patients identified that risk factors for the malignant transformation of endometriosis include a personal history of endometriosis and/or adenomyosis, prior hysterectomy and/or BSO, and HRT use (predominantly estrogen-alone) for longer than 5 years [84]. This review showed that 50% of the patients had endometrioid histology, 10% had clear cell, and 6.7% had both endometrioid and clear cell carcinoma. Furthermore, there was a favorable survival rate, with 12% mortality at a mean follow-up time of 19 months [84]. Similar findings were shown in another systematic review in 2014, showing improved overall survival in patients with EAOC compared with non-EAOC (although this finding was not significant in subgroup analysis, and there was also no difference in progression-free survival between the groups) [85]. Additionally, atypical endometriosis defined as cytological atypia may be a precursor to EAOC [86].

Another systematic review of 48 cases of patients with the malignant transformation of endometriosis of the abdominal wall showed that clear-cell carcinoma occurred in 67% and endometrioid adenocarcinoma occurred in 15% of the cases. The patients were treated with surgical excision, chemotherapy, or radiation, with an overall 5-year survival rate of 40% [87]. The implementation of protective efforts with tubal ligation, salpingectomy, or/and use of oral contraceptive pills are effective for risk reduction in EAOC in the pre and perimenopausal stages. The long-term use of OCPs (10 years or more) has been shown to reduce the risk of ovarian cancers by 80%, and can be useful for EAOC prevention as well [78].

The association of endometriosis with other types of cancer, specifically breast and endometrial uterine cancer, has also been reported. A meta-analysis from 2022 included 14 cohort studies and seven case–control studies which showed an increased risk of endometrial cancer (relative risk 1.662) and breast cancer (1.082), both of which were statistically significant, although the findings are limited by study heterogeneity [88]. The authors suggest that the association may be due to the peripheral conversion of androgens to estrogen within endometriotic tissue, which then caused the stimulation of the breast or endometrium which also have estrogen receptors. This theory has biological plausibility as both breast and endometrial cancers can be caused by excessive estrogen exposure.

## 7. Conclusions and Future Directions

Endometriosis is a common gynecologic condition which can affect women starting in adolescence. Prior studies emphasized that endometriotic lesions ceased activity with the onset of menopause due to hypoestrogenism, and ovarian suppression and/or oophorectomy have historically been a mainstay of treatment. Recent research, including a multitude of case reports, case series, and retrospective studies, have shown that endometriosis remains a diagnostic consideration in postmenopausal women. The etiology is poorly understood due to limited studies on this topic and heterogeneity in clinician management and reporting [3,15,89].

With regard to management, engaging patients in shared decision making empowers them to actively participate in treatment discussions and express their preferences, values, and concerns. Using a combination of physical exam findings, laboratory assessment when appropriate, and imaging, clinicians can provide suggestions and recommendations in a format that is accessible and understandable to patients, enabling them to make informed choices aligned with their values and preferences. Additionally, exploration into the role of immune dysregulation and inflammatory pathways in persistent or de novo endometriosis beyond menopause can identify potential therapeutic targets, opening avenues for innovative diagnostic and treatment modalities.

This article reviews many of the considerations that are relevant to the postmenopausal patient, including a differential diagnosis of pelvic pain, imaging considerations, surgical indications, risk factors for the reactivation or de novo development of endometriosis, and malignant transformation. We hope that additional attention to this topic will stimulate research, including the longitudinal studies of women as they transition to menopause, to follow the natural history of this disease. We also believe that collaborative interdisciplinary research endeavors are key for unraveling the pathophysiology of endometriosis in the postmenopausal population, driving forward evidence-based guidelines, and optimizing clinical outcomes.

## Figures and Tables

**Table 1:** Differential diagnosis of pelvic pain in postmenopausal women.

Condition	Definition	Management
Genitourinary syndrome of menopause	An array of symptoms caused by hypoestrogenic changes to the vulva, vagina, urethra, or bladder that are typically seen in postmenopausal women. Also referred to as vulvovaginal atrophy.	Estrogen vaginal cream, vaginal lubricants, and moisturizers
Pelvic floor tension myalgia	A common type of pelvic pain specific to the pelvic floor. A type of myofascial pain that typically arises from the spasticity, hypersensitivity, and dysfunction of the pelvic floor muscles; fascia/abdominal wall; trigger points; and back pain.	Pelvic floor physical therapy, vaginal suppositories, pelvic floor trigger point injections, and pelvic floor Botox injections
Pudendal neuralgia	Pelvic pain associated with hypersensitivity to the pudendal nerves which can cause nerve entrapment and inflammation. Pain is typically increased with sitting and can be caused by previous pelvic trauma, childbirth, or changes related to age.	Pelvic floor physical therapy, vaginal suppositories, pudendal nerve block, and pudendal neurolysis surgery
Pelvic adhesions	Scar tissue and adhesions in the pelvis from prior surgery or inflammatory conditions such as endometriosis.	Consideration of surgery for the lysis of adhesions (controversial)
Vulvodynia	Vulvar pain, likely idiopathic in nature, that occurs for three months or more. Hypersensitivity to the vulva with sitting, palpation, and with activity.	Estrogen vaginal cream, topical lidocaine, topical neuromodulating medications, and vestibulectomy
Pelvic congestion syndrome	Pelvic pain associated with dilated or engorged pelvic vascular structures. This can also be associated with Nutcracker syndrome and vulvovaginal varicostities.	Progestins, embolization or coiling of abnormal vasculature, and consideration of surgery
Painful bladder syndrome	Chronic bladder pain, also known as interstitial cystitis, which significantly impacts the quality of life in men and women. Etiology is unknown and likely multi-factorial.	Lifestyle modifications and avoidance of bladder irritants, anti-histamine medications, bladder instillations, bladder Botox injections, and bladder hydrodistention
Coccydynia	Chronic coccyx and/or tailbone pain causing tenderness and localized pain to the coccyx primarily with sitting and the supine position. Symptoms may also lead to pelvic floor dysfunction, dyspareunia, pelvic floor tension myalgia, and defecation disorders.	Pelvic floor physical therapy, coccyx trigger point injections, and ganglion impar block
Endometriosis	Endometrial glands and stomas found outside of the endometrial cavity that can cause pelvic pain, dyspareunia, menorrhagia, dysmenorrhea, potential infertility, and chronic pain.	Hormone suppression (OCPs, progestins, annd GnRH analogs), and surgical excision
Adnexal masses	Masses typically seen in the adnexal space or on the ovary; may be cystic lesions or tumors that range from simple to complex, benign to malignant.	Surgical excision
Vulvar dermatoses	Dermatitis to the vulva that is typically inflammatory in nature. Symptoms can include erythema, prurutitis, chronic irritation, and/or vulvar lesions or rash.	Estrogen vaginal cream, topical steroids, vaginal laser therapy, and vaginal platelet-rich plasma (experimental)
Vaginal cuff pain	Persistent tenderness, burning, or discomfort at the center or apexes of the vaginal cuff. This may be related to vaginal adhesions, previous pelvic radiation, or atrophy.	Pelvic floor physical therapy, vaginal suppositories, vaginal cuff trigger point injections, consideration of surgical revision of cuff (controversial)
Ovarian remnant syndrome	Ovarian tissue seen in a patient who previously underwent oophorectomy. This can lead to pelvic pain and adnexal masses.	Surgical excision of the remnant ovarian tissue

**Table 2:** O-RADS scoring system [38,39]. Adapted from algorithms published by the American College of Radiology.

O-RADS Score	Risk Category	U/s Features	MRI Features
**O-RADS 0**	Incomplete evaluation	N/a	N/a
**O-RADS 1**	Normal ovary	**Risk of malignancy: N/a** - No ovarian lesion - Physiologic cyst: follicle or corpus luteum < 3 cm	**Risk of malignancy: N/a** - No ovarian lesion - Physiologic cyst: follicle, hemorrhagic cyst, or corpus luteum < 3 cm
**O-RADS 2**	Almost certainly benign	**Risk of malignancy: <1%** - Simple cyst - Unilocular, smooth, non-simple cyst < 10 cm - Bilocular, smooth cyst < 10 cm - Typical benign ovarian or extra-ovarian lesion < 10 cm	**Risk of malignancy: <0.5%** - Unilocular cyst with any type of fluid content, no wall enhancement, no enhancing solid tissue - Unilocular cyst with endometriotic fluid content; smooth enhancing wall - Lesion with lipid content; no enhancing solid tissue - Lesion with “dark T2/dark DWI” solid tissue - Dilated fallopian tube: simple fluid content; thin, smooth wall/endosalpingeal folds with enhancement - Para-ovarian cyst: any type of fluid; thin, smooth wall +/− enhancement
**O-RADS 3**	Low risk	**Risk of malignancy: 1–10%** - Typical benign ovarian lesion ≥ 10 cm - Uni- or bilocular cyst, smooth, ≥10 cm - Unilocular cyst, irregular, any size - Multilocular cyst, smooth < 10 cm, color score (CS) < 4 - Solid lesion, ±shadowing, smooth, any size, CS = 1 - Solid lesion, shadowing, smooth, any size, CS 2–3	**Risk of malignancy: <5%** - Unilocular cyst: proteinaceous, hemorrhagic, or mucinous fluid content - Multilocular cyst: any type of fluid, no lipid content - Lesion with solid tissue (excluding T2 dark/DWI dark) - Dilated fallopian tube: non-simple fluid and thin wall/folds or simple fluid and thick, smooth wall/folds
**O-RADS 4**	Intermediate risk	**Risk of malignancy: 10–50%** - Bilocular cyst without solid component(s) - Multilocular cyst without solid component(s) - Unilocular cyst with solid component(s) - Bi- or multilocular cyst with solid component(s) - Solid lesion, non-shadowing	**Risk of malignancy: 50%** - Lesion with solid tissue (excluding T2 dark/DWI dark): Intermediate-risk time–intensity curve on dynamic contrast-enhanced (DCE) MRI and any lesion with solid tissue enhancing ≤ myometrium at 30–40 s on non-DCE MRI - Lesion with lipid content containing large volume enhancing solid tissue
**O-RADS 5**	High risk	**Risk of malignancy: ≥50%** - Unilocular cyst, ≥4 pps, any size, any CS - Multilocular cyst with solid component(s), any size, CS 3–4 - Solid lesion, ± shadowing, smooth, any size, CS 4 - Solid lesion, irregular, any size, any CS - Ascites and/or peritoneal nodules	**Risk of malignancy: 90%** - Lesion with solid tissue (excluding T2 dark/DWI dark): High-risk time–intensity curve on DCE MRI, and any lesion with solid tissue enhancing > myometrium at 30–40 s on non-DCE MRI - Peritoneal, mesenteric, or omental nodularity or irregular thickening with or without ascites

**Table 3:** Examples of case reports of de novo postmenopausal endometriosis.

Author	Year	Pathology	Case Description
Vorstman [63]	1983	Benign bladder endometrioma	A 64-year-old female with prior hysterectomy and no current HRT use presented with hematuria and stress urinary incontinence. A cystic mass was noted at the bladder dome and confirmed to be endometriosis with a bladder biopsy. She underwent partial cystectomy, left oophorectomy, and Marshall-Marchetti procedure.
Ismail [64]	1997	Benign ovarian endometrioma	A 52-year-old female with breast cancer (using tamoxifen)and no prior history of endometriosis or HRT use underwent hysterectomy with BSO due to vaginal bleeding. She had a cystic 10 cm ovarian mass.
Kurioka [65]	1999	Benign ovarian endometrioma	A 55-year-old postmenopausal woman without known endometriosis or HRT use presented with a partially solid ovarian mass. She underwent an abdominal hysterectomy with BSO.
Deval [66]	2002	Benign endometriosis	A 69-year-old woman with pelvic pain, constipation, and progressive weight loss with a 15 cm lesion causing compression of the sigmoid colon. There was no prior history of endometriosis or HRT use, and tumor markers were normal. She underwent laparotomy with radical hysterectomy, BSO, colon resection with sigmoid end-colostomy and Harmann’s pouch creation.
Goumenou [67]	2003	Benign endometriosis	A 67-year-old woman with pelvic pain and deep dyspareunia, prior hysterectomy, and she had been using estrogen HRT along with testosterone implants. Imaging showed a 2.9 cm cystic left ovarian mass, with normal Ca-125. Laparoscopy showed left ovarian endometriosis, extensive peritoneal implants, a large retroperitoneal mass, and obliteration of the posterior cul-de-sac.
Popoutchi [68]	2008	Benign rectal endometriosis	A 74-year-old female presented hematochezia and tenesmus; with prior hysterectomy and BSO and no prior HRT use. Imaging revealed a large bowel obstruction, and colonoscopy showed a friable rectal tumor, with pathology consistent with endometriosis. The patient underwent rectosigmoidectomy with protective transversotomy, and ultimately returned for end colostomy due to the stenosing recurrence of the rectum.
Manero [69]	2009	Benign ovarian endometrioma	A 62-year-old-female with no prior history of endometriosis or HRT use presented with pelvic pain. Imaging revealed a 4.4 cm left ovarian mass, with normal tumor markers. Laparoscopic BSO was performed.
Maeda [70]	2009	Benign bladder endometriosis	A 65-year-old woman with painless hematuria and abdominal pain, with prior hysterectomy and no prior HRT use. Imaging showed a vesical polypoid mass, and cystoscopic biopsy and transurethral resection of the mass revealed endometriosis.
Agarwal Sharma [71]	2016	Benign ovarian endometrioma	A 69-year-old woman with abdominal distention and short-term leg swelling. Ca-125 was elevated to 120 u/mL, and imaging showed a 25 cm cystic pelvic mass which was suggestive of malignancy with peritoneal carcinomatosis. She underwent exploratory laparotomy with cytoreduction, and findings showed diffuse endometriosis along the peritoneal surfaces in addition to the large ovarian mass.
Ianieri [72]	2017	Benign endometriosis	A 63-year-old female with abdominal pain and limb swelling, who was noted to have a retroperitoneal mass causing deep vein thrombosis because of the extrinsic compression of the left iliac vein. Laparotomy with endometriosis excision was performed.
Solima [73]	2019	Benign endometriosis	A 60-year-old postmenopausal woman with no prior HRT use, and no known endometriosis or chronic pelvic pain presented with a rectovaginal mass. Imaging revealed a mass involving the uterus, posterior bladder wall, and rectum, and a cystic lesion was seen in the bladder trigone during cystoscopy. She underwent laparoscopic BSO and bladder biopsy.
Naem [74]	2020	Benign abdominal endometrioma	A 67-year-old woman presented with bowel obstruction and right-sided hydronephrosis in the setting of a 17 × 26 cm abdominal mass. The patient underwent laparotomy with the en-bloc resection of the mass.
Devasilpa [75]	2022	Benign ovarian endometrioma	A 52-year-old postmenopausal patient presented with abdominal distention, and imaging showed a 30 × 13 × 20 cm right ovarian cystic mass. Ca-125 was normal and she was not previously using HRT. Laparotomy with right salpingo-oophorectomy was performed, with extrusion of 5 L of chocolate cyst fluid.
Zografou [76]	2023	Benign ovarian endometrioma	A 60-year-old previously healthy female presented with a 26 cm ovarian mass. Pre-operative Ca-125 was 512.9 U/mL, and all other labs were normal. The patient underwent laparotomy with BSO.
